# Numerical Analysis of the Effect of Nanoparticles Size and Shape on the Efficiency of a Micro Heatsink

**DOI:** 10.3390/nano12213836

**Published:** 2022-10-30

**Authors:** Saeed Alqaed, Jawed Mustafa, Fahad Awjah Almehmadi, Mathkar A. Alharthi, Mohsen Sharifpur, Goshtasp Cheraghian

**Affiliations:** 1 Mechanical Engineering Department, College of Engineering, Najran University, P.O. Box 1988, Najran 61441, Saudi Arabia; 2 Department of Applied Mechanical Engineering, College of Applied Engineering, Muzahimiyah Branch, King Saud University, P.O. Box 800, Riyadh 11421, Saudi Arabia; 3 Department of Chemical Engineering, College of Engineering at Yanbu, Taibah University, Yanbu 41911, Saudi Arabia; 4 Department of Mechanical and Aeronautical Engineering, University of Pretoria, Pretoria 0002, South Africa; 5 Department of Medical Research, China Medical University Hospital, China Medical University, Taichung 404, Taiwan; 6 Institut für Chemie and IRIS Adlershof, Humboldt-Universität zu Berlin, 10117 Berlin, Germany; 7 Department of Chemistry, King’s College London, London WC2R 2LS, UK

**Keywords:** nanoparticle shape, micro heat sink, efficiency, economy

## Abstract

In this paper, two novel micro heat sinks (MHSs) were designed and subjected to thermal analysis using a numerical method. The fluid used was Boehmite alumina–water nanofluid (NFs) with high volume fractions (VOFs). Studies were conducted to determine the influence of a variety of nanoparticle (NP) shapes, such as platelet brick, blade, cylinder, and Os. The heatsink (HS) was made of copper, and the NFs entered it through the middle and exited via four outlets at the side of the HS. The finite element method was used to simulate the NFs flow and heat transfer in the HSs. For this purpose, Multi Physics COMSOL software was used. The maximum and middle values of HS temperature (T-MAX and T-Mid), thermal resistance (TH-R), heat transfer coefficient (h), FOM, etc., were studied for different NP shapes, and with Reynolds numbers (Re) of 300, 1000, and 1700, and VOFs of 0, 3, and 6%. One of the important outcomes of this work was the better thermal efficiency of the HS with rectangular fins. Moreover, it was discovered that a rise in Re increased the heat transfer. In general, adding NPs with high VOFs to MHSs is not appropriate in terms of heat. The Os shape was the best NP shape, and the platelet shape was the worst NP shape for high NPVOF. When NPs were added to an MHS, the temperature of the MHS dropped by an average of 2.8 or 2.19 K, depending on the form of the pin-fins contained inside the MHS (circular or square). The addition of NPs in the MHS with circular and square pin-fins enhanced the pressure drop by 13.5% and 13.3%, respectively, when the Re = 1700.

## 1. Introduction

Micro heat sinks, also known as MHSs, are extensively utilized in electronic equipment and may be found in a broad variety of common electronics. Heat sinks (HSs) are responsible for cooling electronic parts, which heat up considerably when operating [1]. They are used to cool devices such as LEDs [2,3,4,5], solar cells [6,7,8,9,10,11], CPUs [12,13,14], etc. Many electronic devices that need cooling have very small or micro-scale dimensions [15,16,17]. Hence, MHSs are used to cool them [18,19,20,21,22]. The HS cooling fluids and the cooling methods differ in different devices: some are cooled via natural convection [23,24,25], and some via forced convection [26,27,28,29]. Fluids such as air [30,31], water [32,33,34,35], oil [36,37], and, most importantly, NFs are utilized as the working fluid in MHSs [38]. In one paper [39], Leela Vinodhan and Rajan designed a new MHS and examined its thermal efficiency. This MHS had four fluid inlets and outlets. The findings of this research showed that raising the fluid PP in the HS brought about an increase in the Nusselt number, while simultaneously bringing about a reduction in the TH-R of the MHS. In another study by Hernandez-Perez et al. [40], the authors examined the effect of placing a heatsink on a solar panel. Their findings showed that heatsinks could reduce panel temperatures by up to 5.1 °C. One of the major challenges in the field of heat transfer using fluids in various heat exchangers is the low thermal conductivity of fluids [41,42,43]. Researchers have tried to increase the thermal conductivity of fluids [44,45,46,47,48]. One of the methods that many researchers have used is to use nanofluids instead of ordinary fluids in heat exchangers [49]. A nanofluid is a stable mixture of nanoparticles in a base fluid [50,51,52,53,54,55,56]. According to various studies, nanofluids have a higher thermal conductivity than normal fluids and, in many cases, can have a better heat exchange than normal fluids [57]. In the last two decades, NFs have been used as the working fluid by many researchers [58,59,60]. Cavities [56,61], solar collectors [43,62], and heat exchangers [63,64,65] are among these devices [66]. Many researchers have used NFs as the working fluid in HSs [67,68]. The NPs used in NFs can have different shapes. Most researchers have studied spherical NPs, but there are other NP shapes with special properties, and their shapes affect their thermal conductivity and viscosity. Some researchers have investigated the effect of the shape of NPs on the thermal efficiency of the studied device [62,69,70]. In one of these studies, Yan et al. [69] examined the effect of using different shapes of Nps in a heat transfer cavity. According to the findings, the use of plate NPs resulted in the lowest possible levels of heat transfer. According to their report, the explanation for this is that adding NPs to the base fluid causes significant changes in viscosity, which in turn cause changes in the thermal conductivity of the fluid. As the surface of the NPs, the form of the NPs has an effect on heat transfer. Bahiraei et al. [71] studied the irreversibility of alumina–water NFs in an MHS with distinct NP forms in one of these experiments. In another study, Arani et al. [72] numerically investigated the shape effect of Boehmite alumina NPs in an ethylene glycol-water fluid in a wavy mini-channel. Their findings indicated that spherical NPs produced the largest Nusselt number (Nu) in the mini-channel. A challenge in electronic industries is the overheating of components such as the CPU. Therefore, researchers have attempted to find HSs with a better heat dissipation capability at a lower PP by changing the geometry of MHSs. 

Since the use of NFs in MHSs has become common, it is necessary to study the effect of using NPs with different shapes in MHSs, as well as those having minute dimensions. Accordingly, this paper involves the thermal analyses of two innovative MHSs. An alumina–water NFs stream flowed inside an MHS, in which the effect of five types of nanoparticle shape were studied. In conclusion, the thermal efficiency of modifying Re, as well as the volume percentage of NPs with various forms was investigated in both HSs. In addition, the FOM index was studied, to examine the effect of adding NPs on the overall efficiency of the HS. Finally, the innovation of the present work can be expressed in the innovative geometry of the heatsink and also the study of its thermal efficiency using nanofluids with different shapes of nanoparticle.

## 2. Problem Statement

**Figure 1:**
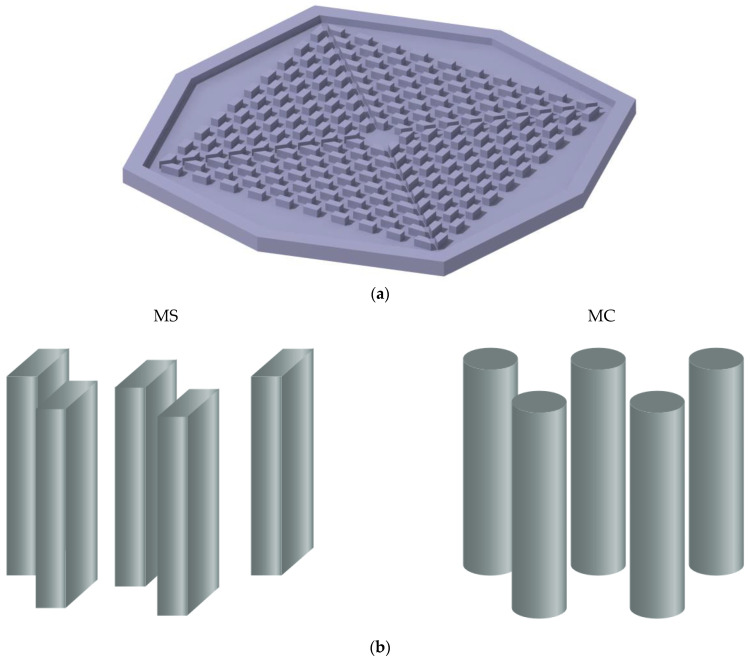

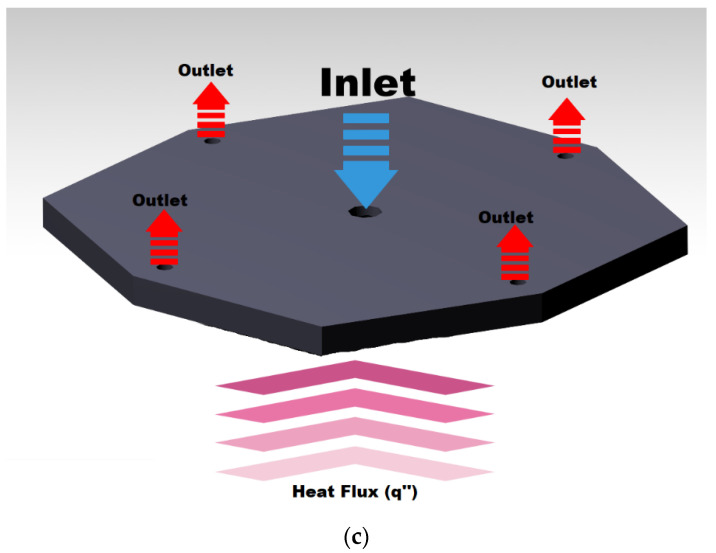
(**a**) Overall schematic and size details of the HS under study. (**b**) Details of the geometry under study. (**c**) The boundary condition applied to the problem geometry.

As can be seen in Figure 1, the MHS has an octagonal shape and is constructed of copper. The MHS under study has one inlet in the middle of the HS cover. It also has four outlets around the HS, which are located on the cover. The details of the HS size are displayed in Figure 1a. The new HS has two types, and fins with rectangular and circular cross-sections were used in them. The corresponding details for both HSs are shown in Figure 1b. The Boehmite alumina–water NFs flow enters the HS in a steady-state and laminar manner. Moreover, the NFs were considered homogeneous and incompressible. The boundary conditions are shown in Figure 1c. The volume percentage of nanoparticles used in the nanofluid in the heatsink is always equal to 6%, which includes different shapes of nanoparticle.

## 3. Governing Equations

NFs flow equations (Equations (1)–(3)) and the energy conservation equation for the solid part of the HS (4) are given in the following [73]:

(1) $$\nabla \cdot \left(\rho_{nf}v\right)=0$$

(2) $$\nabla \cdot \left(\rho_{\mathrm{nf}}vv\right)=-\nabla P+\nabla \cdot \left(\mu_{\mathrm{nf}}\nabla v\right)$$

(3) $$\nabla \cdot \left(\rho_{\mathrm{nf}}vc_{p,\mathrm{nf}}T\right)=\nabla \cdot \left(k_{\mathrm{nf}}\nabla T\right)$$

(4) $$\nabla \cdot \left(k^{*}\nabla T\right)=0$$

### 3.1. Thermal Efficiency

The coefficient of heat transfer is one of the most critical parameters in the study of thermal efficiency. The convection heat transfer coefficient can be measured as follows [73]:

(5) $$h=\frac{q^{\prime \prime }}{T_{\mathrm{Mid}}-T_{\mathrm{ave}}}$$

In the above equation, T_ave_ denotes the average of the inlet and outlet temperatures of the HS and is defined in the form of $\frac{T_{\mathrm{out}}-T_{\mathrm{in}}}{2}$. $T_{\mathrm{Mid}}$ is the average temperature of the constant-flux section of the HS, and $q^{\prime \prime }$ represents the heat flux applied from the microchip to the HS. The figure of merit (FOM) comprehensively shows the effect of NF addition on the heat transfer coefficient and pressure difference. This parameter indicates whether the addition of nanoparticles positively affects the HS. This relationship is given in the following:

(6) FOM=hnfhf∆Pnf∆Pf

The TH-R of the HS and the temperature uniformity of its bottom section are defined as follows, respectively. These two parameters are significant in the study of thermal efficiency and have been investigated in most papers about HSs.

(7) $$R=\frac{T_{\mathrm{Mid}}-T_{\mathrm{in}}}{q^{\prime \prime }}$$

(8) $$\theta =\frac{T_{\mathrm{Max}}-T_{\min}}{q^{\prime \prime }}\;$$

The subscripts Max and Min on the bottom of the HS show the maximum and minimum temperatures.

### 3.2. NFs Properties

The density and basic heat potential of the NFs were calculated using the following relationships:

(9) $$\rho_{\mathrm{nf}}={\varphi \rho }_{\mathrm{np}}+\left(1-\varphi \right)\rho_{f}$$

(10) $$c_{p,\mathrm{nf}}=\frac{\left(1-\varphi \right){\left({\rho c}_{p}\right)}_{f}+\varphi {\left({\rho c}_{p}\right)}_{np}}{\rho_{\mathrm{nf}}}$$

In the above equations, the subscript p denotes the NPs, and the subscript f denotes water. Moreover, φ represents the volume fraction of the NPs. To examine the effect of the NP shape using the paper by Timofeeva et al. [74], the impact of various NP shapes was studied for Boehmite alumina NPs. The following are the relationships that were produced in this research with regard to the thermal conductivity and viscosity of the NFs:

(11) $$\frac{k_{f}}{k_{\mathrm{bf}}}=1+C_{k}\varphi$$

(12) $$\frac{\mu_{f}}{\mu_{\mathrm{bf}}}=1+A_{1}\varphi +A_{2}\varphi^{2}$$

$A_{1}$ and $A_{2}$ are given in Table 1.

The thermal conductivity of the Os-shaped NPs can be calculated using the following relationship: [75].

(13) $$\frac{k_{\mathrm{nf}}}{k_{f}}=\frac{k_{np}+\left(n-1\right)k_{f}-\left(n-1\right)\left(k_{f}-k_{np}\right)\varphi }{k_{np}+\left(n-1\right)k_{f}+\left(k_{f}-k_{np}\right)\varphi }$$

In the above relationship, the parameter n denotes the shape factor and is expressed in the form n=ψ/3. Table 2 provides the value for the parameter ψ that describes the Os-shaped NPs.

The following equation can be employed for the viscosity of the NFs with Os-shaped NPs [77].

(14) $$\frac{\mu_{\mathrm{nf}}}{\mu_{f}}={\left(1-\frac{\varphi }{\varphi_{m}}\right)}^{-2}$$

The parameter $\varphi_{m}$ for the Os NPs is shown in Table 2, according to Muller et al. [68]. The parameter δ, which denotes the geometrical characteristic of the Os-shaped NPs, is as follows [78]:

(15) $$\delta =\frac{c}{a}$$

Table 3 illustrates the characteristics of both water and the Boehmite alumina NP, as well as the various NPs’ three-dimensional shapes.

### 3.3. Boundary Conditions

The NFs that have a boundary condition at constant velocity and a temperature of 293.15 K enter the HS through the central portion with a diameter of 4 mm. Following their journey through the HS, the NFs emerge from the outlets, where they encounter the boundary condition of continuous pressure. There is no discernible variation in the pressure between this location and the pressure of the surroundings. The base of the HS as a whole is subjected to a constant heat flow that averages 88,000 W/m^2^ throughout its whole. Figure 1c provides more information on the HS size. The outer sections of the HS are all insulated, and there is no temperature jump or slip on the walls.

## 4. The Numerical Solution, Validation, and Mesh-Independence of the Solution

A numerical technique that is based on the idea of finite elements was used to investigate and study this subject. The four processes that comprise the finite element process are, in order, the creation of the variational formulation, the selection of the discretization method, the selection of the solution algorithm, and lastly, the acquisition of the results. In the first phase of the process, the equations are rewritten using a weak formulation, so that they are in the integral form rather than the differential form. Conversion to the integral form can be accomplished by a variety of approaches. In this particular study, the Galerkin approach was used. Creating the numerical mesh, calculating the shape function for the mesh elements, and projecting the reference elements onto the mesh elements are the three parts that make up the equation discretization approach.

In this particular study, the solution domain was meshed using components that were octagonal in shape.

In addition, utilizing first- and second-order form functions, each component of the fluid and solid media, as well as the influence that they have on the solution, were investigated. In the third phase, the equation scheme that was produced from the discretization was solved with the help of a method called direct solution. Finally, the consequences of the solution that were ideal for the situation were determined. All of the simulations were carried out on a desktop computer that had 16 gigabytes of memory and that was powered by an Intel Core i7 processor operating at a speed of 4 GHz. The simulation took an average of 120 min, and a total of 81 versions were simulated.

Using unstructured meshing of octagonal cells, the problem geometry was discretized. The finite element approach often employs octagonal cells for meshing. A shape feature was used for each cell as the relation between the nodes and the values within each cell. First- and higher-order polynomials were used to express these functions. It was determined that the solution was not reliant on the computational mesh by comparing the results obtained from a number of different computational meshes after the solution had been found for a particular domain, via the use of several meshes of first- and second-order form functions. The amount of cells that make up each mesh is listed in Table 4. The final solution for Mesh No. 5 was achieved using a second-order form function. As a result, the errors of the other implementations were compared using this value.

**Figure 2:**
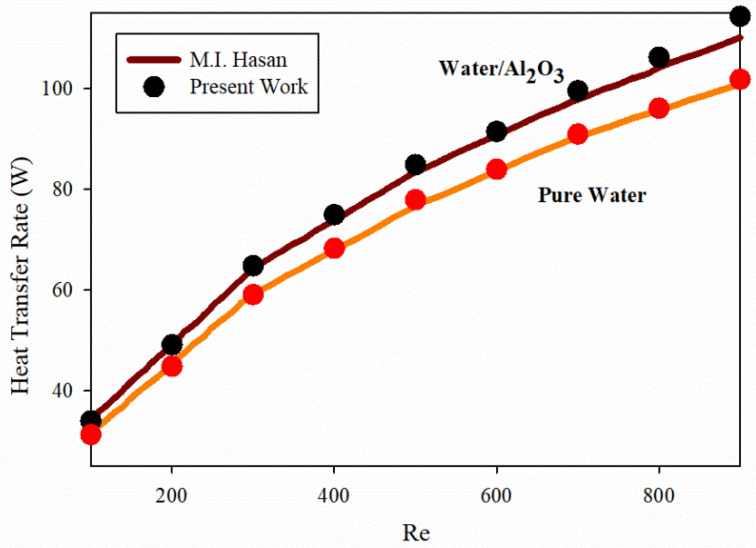
The convective heat transfer coefficient was compared with a previous work and that of [79] for a variety of Re for both NFs and pure fluid in this study.

The simulation effects of cooling a pin fin of an HS using circular pin fins in a work by Hassan [79] were compared to the present work in the second comparison. As seen in Figure 2, the average error of the observed simulation was 3.47 percent.

## 5. Results and Discussion

Table 5 displays the thermal conductivity coefficient of the NFs with various NP forms at a 6% volume fraction, as well as its increase over time compared to the base fluid. The highest increase in thermal conductivity was seen for the Os-shaped NPs, while the lowest increase in thermal conductivity was seen for the platelet-shaped NPs.

Table 6 shows the viscosity of the NFs with different NP shapes at a 6% volume fraction and its increase concerning the base fluid. It can be observed that the smallest rise in viscosity belonged to the Os-shaped NPs, and the most significant increase corresponded to the platelet-shaped NPs. A comparison of these two tables reveals that adding NPs at high VOFs, especially for shapes other than Os, slightly increased the thermal conductivity but increased the viscosity several fold.

**Figure 3:**
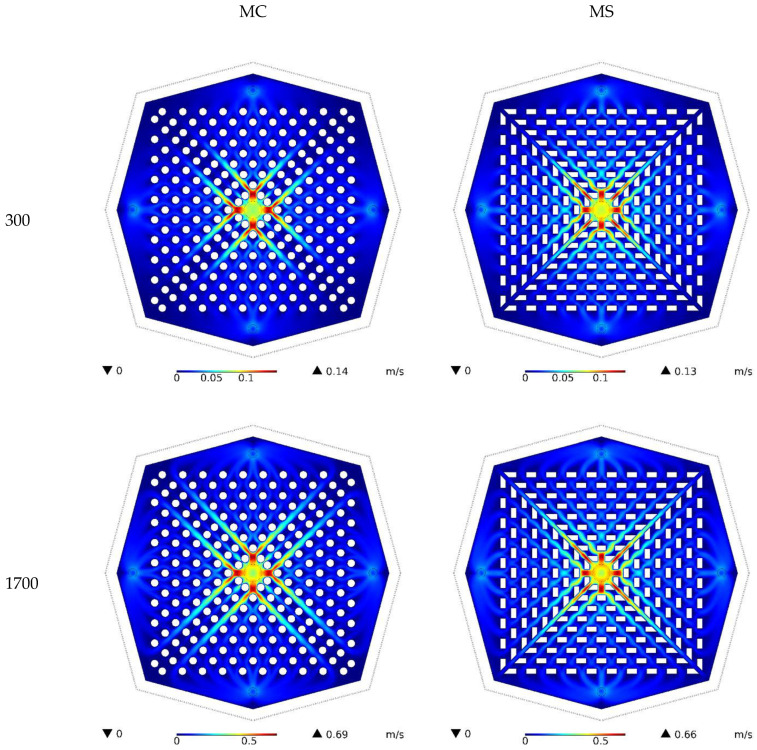
Water velocity contours for both types of HS at Reynolds numbers of 300 and 1700.

Figure 3 depicts the velocity contours for both kinds of HS for Re values of 300 and 1700, for the medium of water. It can be noticed that a rise in Re resulted in a significant increase in the highest possible fluid velocity in the HS. Given the constant fluid properties and fluid inlet diameter, an increase in Re translated to an increase in fluid velocity. Therefore, the fluid enters the HS at a higher velocity and, hence, its maximum velocity increases. The MC type had a larger maximum velocity than the two HS types. The fluid velocity decreased with the motion of the fluid toward the HS sides and an with increase in its cross-section, such that the fluid velocity was almost constant around the HS.

**Figure 4:**
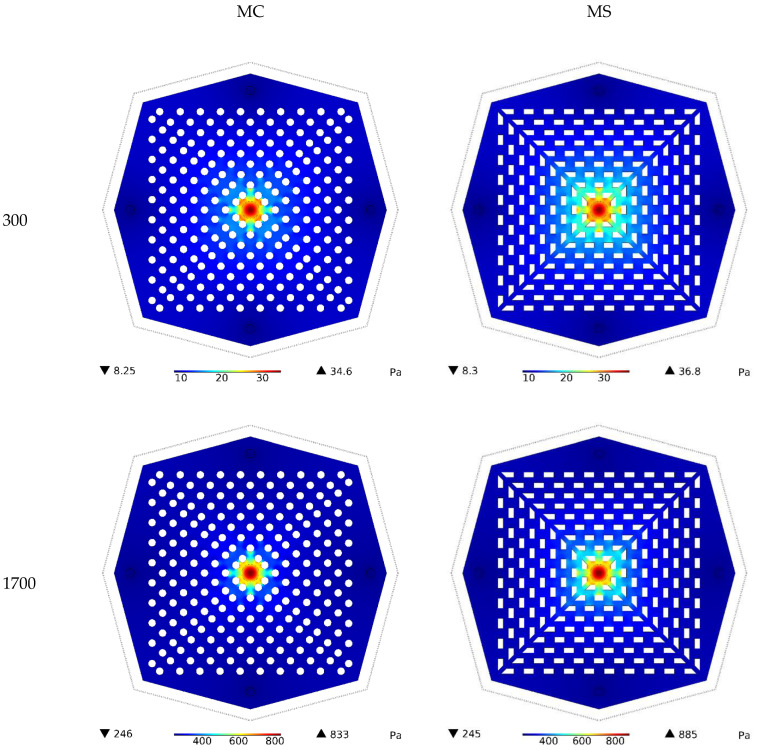
Pressure contours for both types of HS at Reynolds numbers of 300 and 1700 for water.

The pressure contours for both kinds of HS at Re values of 300 and 1700 for water are shown in Figure 4. It can be seen that a rise in Re resulted in a noticeable increase in the number of pressure changes. Given the constant value of pressure at the HS outlets, which is equal to atmospheric pressure, it is the positive pressure created at the inlet that drives the fluid. Therefore, the higher the fluid velocity desired, the larger the pressure required at the inlet. Hence, the pressure difference caused a higher increase in velocity. Moreover, the pressure drop created in the HS affected the pressure difference, and it was observed that the pressure difference created in the MS heat sink was higher than that in the other type, since the pressure drop in this model increased due to sharp corners.

**Figure 5:**
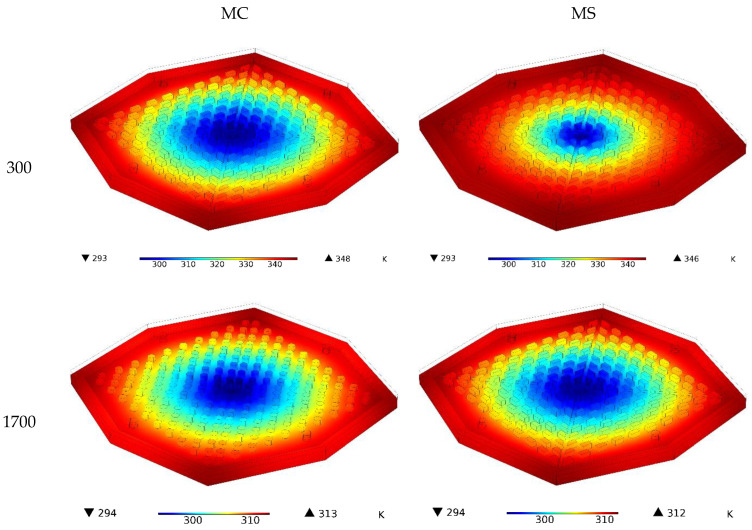
Three-dimensional temperature contours for the two HSs for different Re for water.

The three-dimensional temperature contours for the two HSs with different Re are shown in Figure 5. It can be seen that the rise in Re and, as a direct consequence of that rise, the increase in fluid velocity had a significant impact on the T-MAX, and as a consequence of this effect, they caused the T-MAX to drop by a significant amount. An increase in the fluid velocity caused the fluid entering the inlet at a low temperature to pass the HS faster and heat up less. Therefore, the fluid at a lower temperature touched the sides of the HS, leading to a better heat transfer. Hence, specifically, the T-MAX of the HS was considerably reduced. Of the two models, the MS model performed better thermally at both Re and had a smaller T-MAX.

**Figure 6:**
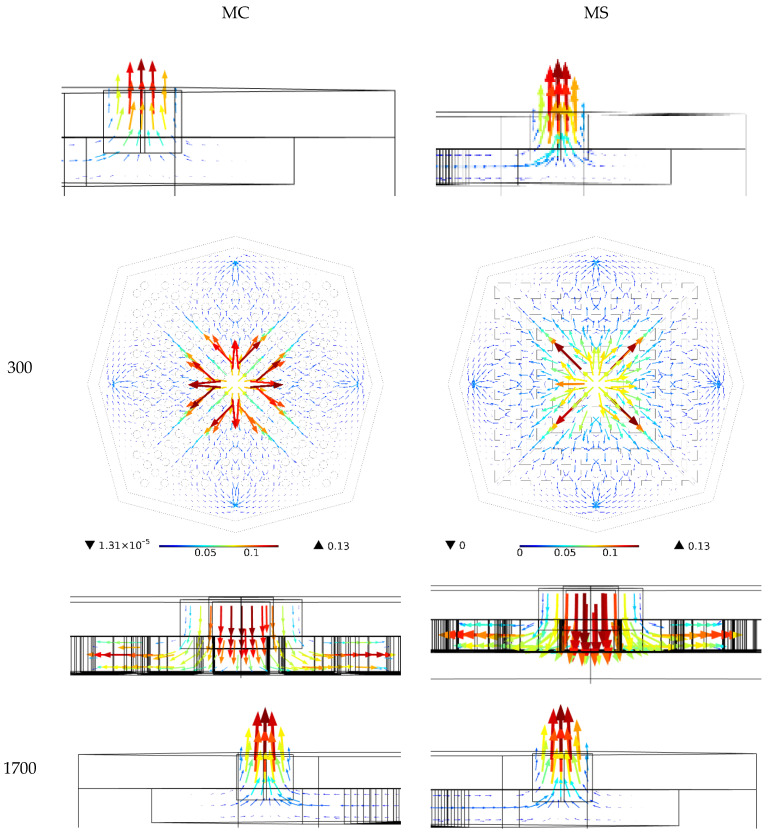

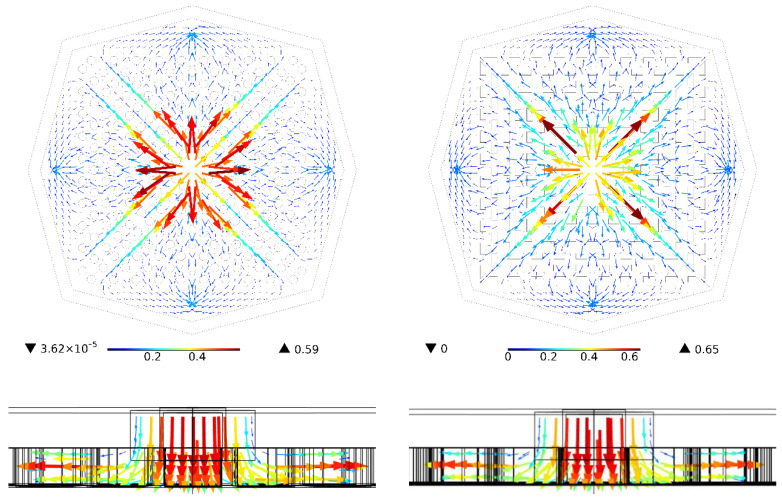
Fluid velocity vector in both HSs with water at a Re of 300 and 1700.

The fluid velocity for both types of HS for water with Re values of 300 and 1700 can be found in Figure 6. The vectors at the inlet and outlet are clearly shown in this figure, and the colors represent the velocities. Here, it is also seen that the rise in Re increased the velocity values in the HS. A comparison of the two kinds of heat sinks reveals that the fluid in the MC heat sink travels toward the outlets more than it goes toward the center, and the fluid flow is greater along this direction. However, the fluid mostly moves toward the corners in the MS heat sink. Since the highest temperature in HSs usually occurs at the corners, the MS heat sink can cool these areas better. Thus, a lower T-MAX occurs in it. It is also observed that the highest velocity occurs in the middle of the inlet and, also, at the middle section of the HS between the bottom and the cover. This is due to the lack of slip on the walls, which causes such a fluid profile.

**Figure 7:**
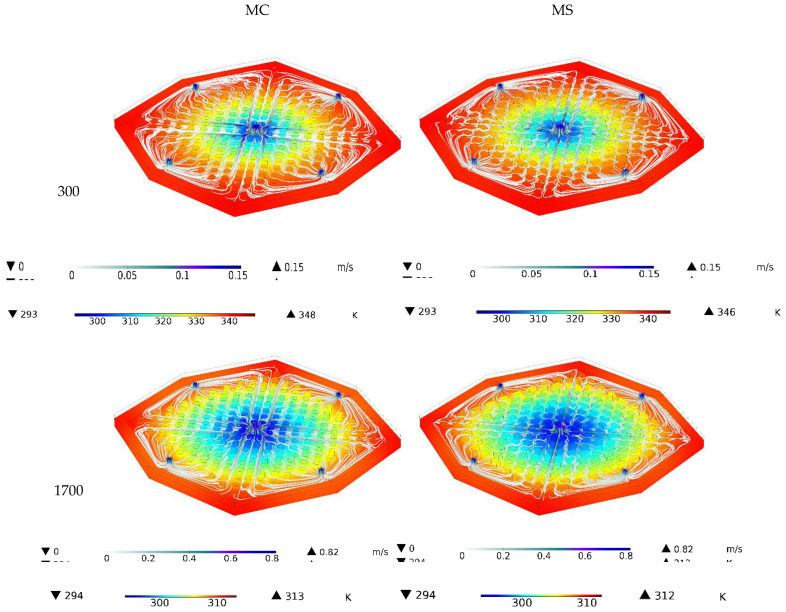
Three-dimensional temperature contours and streamlines in the heatsink for two different Reynolds numbers and two types of heatsink for water.

Figure 7 demonstrates the three-dimensional temperature contours and streamlines in the heatsink for two different Reynolds numbers and two types of heatsink for water. In both heatsinks, the water flow was specified from inlet to outlet. It can be seen that the temperature of the heatsink was reduced with the fluid velocity inside the heatsink. The middle of the heatsink, where the fluid collides vertically, was the coldest part of the heatsink, and the sides of the heatsink are the warmest surfaces. Considering the color of the streamlines, the maximum velocity was observed at the heatsink inlet and outlet.

**Figure 8:**
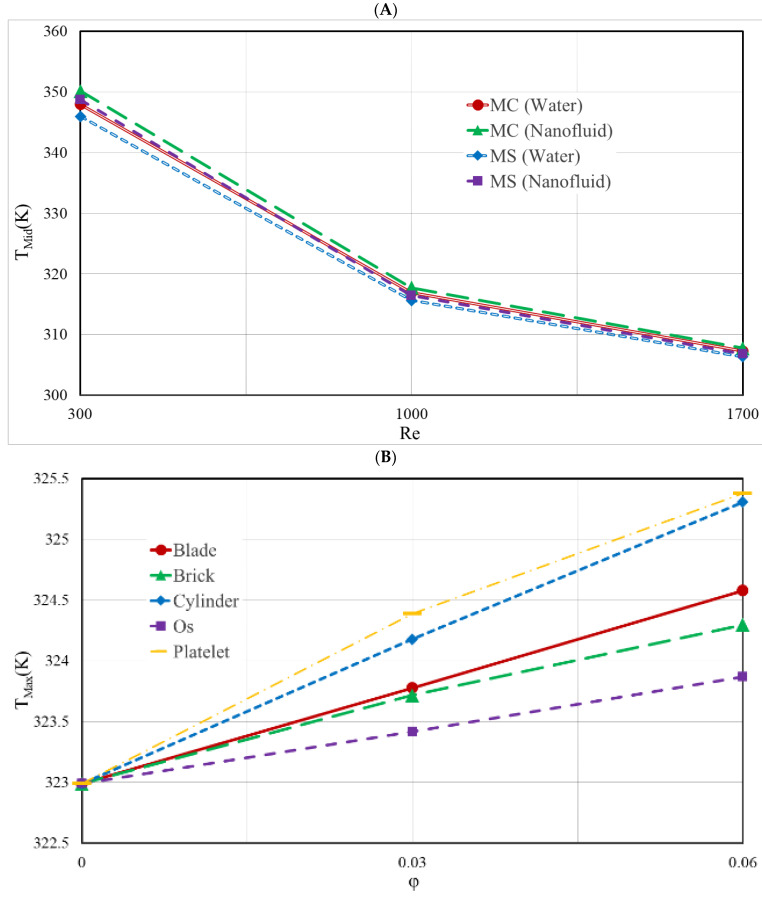
(**A**) the T-MAX for two different types of HS for various Re; (**B**) the average temperature at the bottom of the HS for different NPVOF with varied forms for the MS heat sink at a Re of 1000.

Figure 8A depicts the T-MAX for two different kinds of HS for various Re, and Figure 8B illustrates the average temperature at the bottom of the HS for various NPVOFs with diverse shapes for the MS heat sink at a Re of 1000. Both of these figures are for the MS heat sink. It can be observed that there was a decrease in T-MAX as a result of the increase in Re. The reason for this is the increase in Re causes a rise in the fluid velocity, which in turn generates a cooler fluid flow throughout the whole of the HS. This is the chain of events that leads to this result. The MS-type HS may be thought of as a T-MAX that has been shrunk down a little. As a consequence of the incorporation of NPs, the T-MAX experiences a slight elevation. The inclusion of NPs also resulted in an increase in the average temperature, with the platelet-shaped NP being responsible for the highest temperature and the Os-shaped NP being responsible for the lowest temperature. According to the table, the addition of NPs resulted in a moderate increase in the thermal conductivity, but a significant reduction in the viscosity of the fluid. A strong increase in the viscosity weakened the flow in the HS and ultimately reduced the heat transfer. Therefore, an increase in temperature was observed with the addition of NPs of different shapes. It is interesting to notice that, even when the Re = 300, when the flow rate in the heatsink was extremely low, the temperature of the heatsink was still lower than 350 K. This is a significant difference. When there is a higher Re, the temperature of the heatsink drops significantly, which is an indication of the heatsink’s capacity to cool.

**Figure 9:**
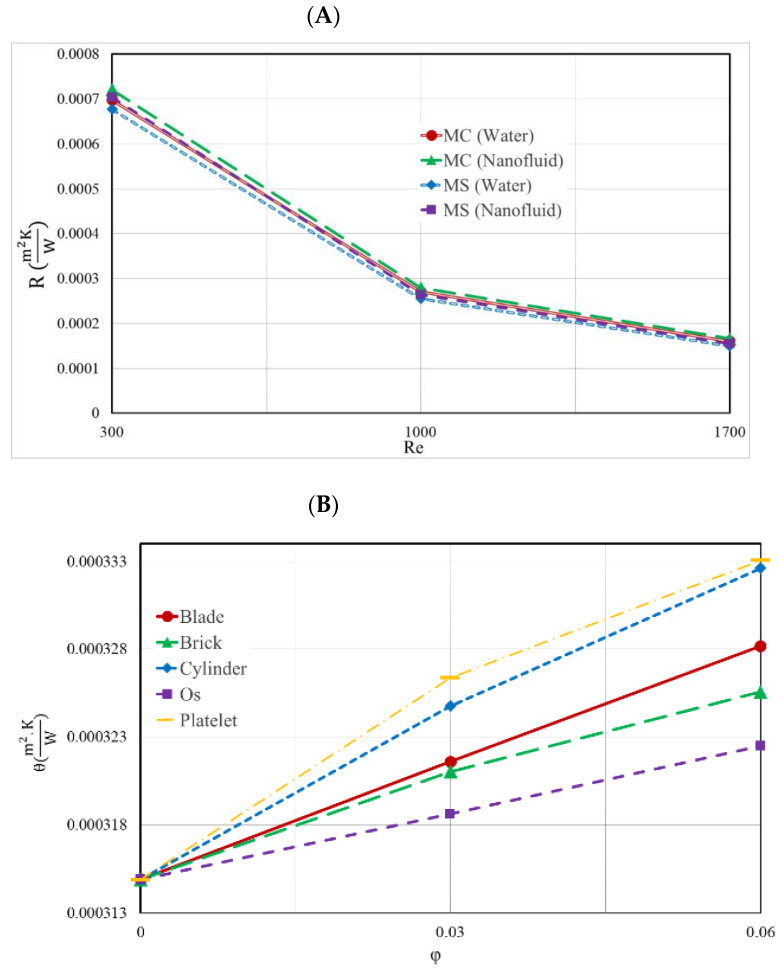
(**A**) TH-R of the two distinct forms of heat sinks for each of the different Re; (**B**) temperature uniformity at the bottom of the MS heat sink for a variety of NPVOFs with varying shapes at a Re of 1000.

Figure 9A illustrates the TH-R of the two HS types for various values of Re, and Figure 9B illustrates the temperature uniformity at the bottom of the MS heat sink for a variety of NPVOFs and shapes, when the Re value was set to 1000. Both of these figures can be found in the accompanying figures. Both of these illustrations may be found in the appendices that accompany the report. When the Reynolds number was increased, the temperature of the heatsink lowered, and its resistance to heat was also lowered as a result of this change. The other kind of heat sink had a TH-R that was much higher than that of the MS heat sink, which had a much lower TH-R. Due to the relative rise in temperature that occurred as a result of the addition of NPs, the TH-R of the HS showed a minor increase as a result of this modification. In addition, the temperature uniformity of the HS was made less consistent when NPs of different forms were added to it. The primary explanation for this is the rise in T-MAX that resulted from the addition of NPs. The worst performance in this regard was that of the platelet-shaped NPs, and the best temperature uniformity belonged to the Os-shaped NPs. The greater the number of NPs, the lower the temperature uniformity.

**Figure 10:**
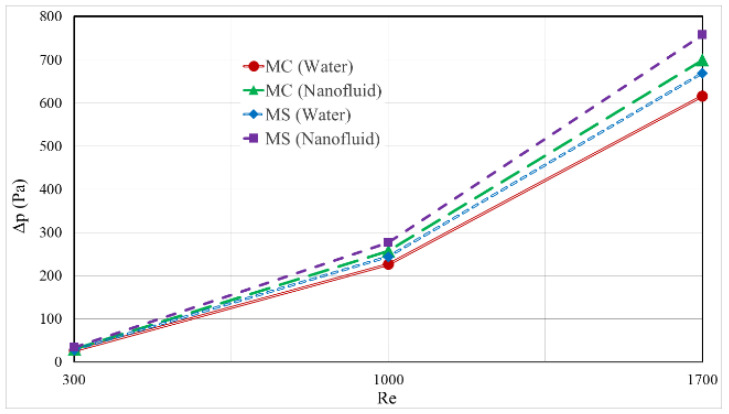
Difference in pressure between the entrance and outflow of the two distinct kinds of HS for 6% Os NFs and water at various Reynolds numbers.

Figure 10 depicts the difference in pressure that occurred between the intake and the outflow of the two HS forms for various Re values with 6 percent Os NFs and water. The increase in Re resulted in a greater pressure disparity between the two HSs. Since the velocity increase caused more losses and a pressure drop along with the HS, the higher Re increased the pressure difference between the inlet and outlet of the HS. The pressure drop was larger in the MS heat sink, due to sharp corners in the fins, which increased the losses and pressure drop. The pressure drop was lower for the circular fins, due to the smoothness of the fin surface. Adding NPs caused an intense increase in losses and a rise in shear stress in the HS, due to the strong increase in viscosity, leading to a significant increase in the pressure drop in the HS.

**Figure 11:**
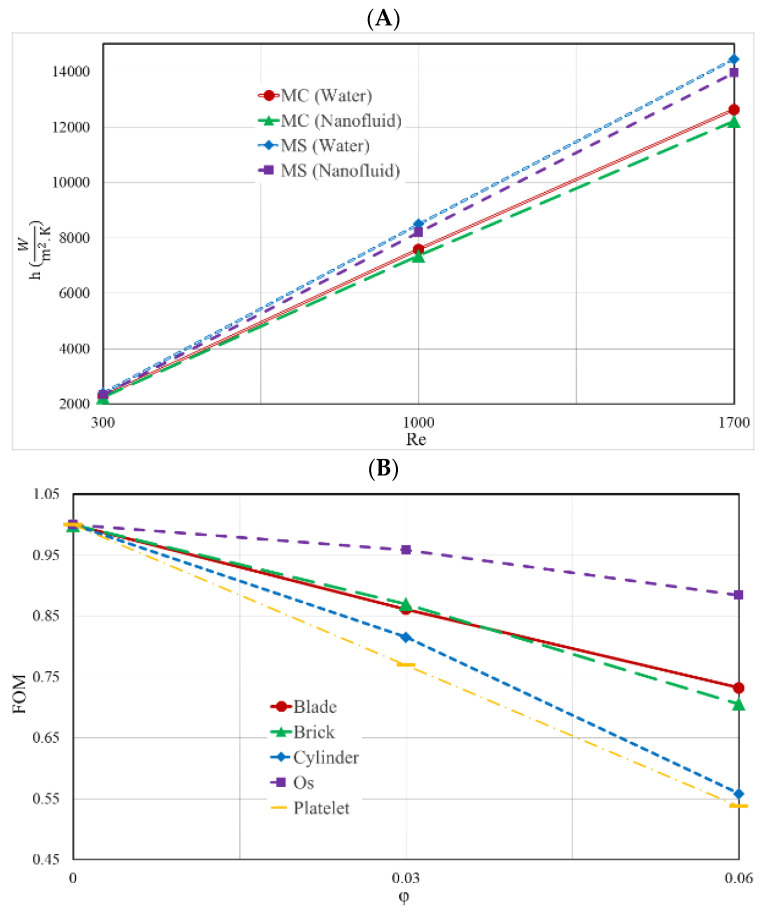
(**A**) heat transfer coefficient of the two distinct HS types for different Re; (**B**) the FOM at the bottom of the MS heat sink at various NPVOFs with variable shapes at a Re of 1000.

Figure 11A depicts the heat transfer coefficient of the two HS types for various Re, and Figure 11B depicts the FOM at the bottom of the MS heat sink at various volumes of NPVOFs with varied shapes at a Re of 1000. Both figures can be found in the figures section of this article. The rise in Re was the result of an increase in the temperature differential, and the decrease in the thickness of the boundary layer was responsible for an increase in the heat transfer coefficient in both types of HS. This rise was shown most prominently in the MS-type of HS. Owing to the greater pressure drop in the HS, this model offered superior heat transfer capabilities, as a result of the increased contact area between the fluid, the heat sink (HS), and the fins. In addition, the better direction of the fluid toward higher-temperature regions improved the heat transfer. Here, it is also observed that adding NPs was not appropriate and reduced the heat transfer coefficient. Adding NPs to water resulted in a descending trend in FOM. The reason for this was the reduction in the heat transfer coefficient with the addition of NPs, on one hand, and the strong increase in the PP, on the other hand, which strongly decreased this index. The usage of NPs with a platelet shape resulted in the FOM being in its worst condition, whereas the use of NPs with an Os shape resulted in the FOM being in the best state. Given the constant flow rate for each condition, the addition of NPs simultaneously resulted in more electricity consumption and a lower heat transfer, which is not economically appropriate.

## 6. Conclusions

In this numerical work, two innovative HSs were designed and simulated. A water-–NF flow enters the HS from the middle and exits it from four outlets at its sides. Boehmite alumina NFs with different NP shapes were used to cool the HSs. The values of thermal efficiency were investigated, and the following findings were reached as a consequence of varying the VOFs of Re and NP:

1. The MS heat sink, in comparison to the other heat sink, generally had superior thermal qualities. It had a lower T-MAX, a smaller TH-R, and greater temperature uniformity. Adding nanoparticles to the heatsink with circular and square pin-fins reduced the average temperature of the heatsink by 2.8 and 2.9 K, respectively when Re = 300.
2. An increase in Re further cooled down the HS and reduced its TH-R, leading to better temperature uniformity in the HSs.
3. In thermal terms, the addition of NPs with a variety of forms is inappropriate for a HS. This is particularly true for large VOFs. The addition of nanoparticles in the heatsink with circular and square pin-fins enhanced the pressure drop by 13.5% and 13.3%, respectively, when the Re = 1700.
4. The platelet NPs had the worst performance in terms of heat transfer when compared to the other NPs shapes. The Os form achieved the greatest levels of performance across the board, with regard to these criteria.
5. A decrease in the FOM may be achieved by including NPs of varying shapes into the MHS in large VOFs.

## Figures and Tables

**Table 1:** Constants may have varying values, depending on the geometry of the NP [74].

	$C_{k}$	$A_{1}$	$A_{2}$
Platelets 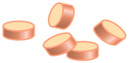	2.61	37.1	612.6
Blades 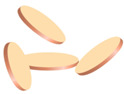	2.74	14.6	123.3
Cylinders 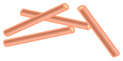	3.95	13.5	904.4
Bricks 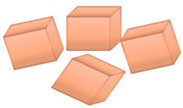	3.37	1.9	471.4

**Table 2:** Constants of different types for the Os-shaped NPs investigated in this research [76].

Case	ψ	δ	$\varphi_{m}$
Os nanoparticles 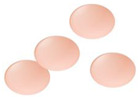	$49.04\times 10^{-2}$	$13\times 10^{-2}$	$57.5\times 10^{-2}$

**Table 3:** The thermophysical characteristics of base fluid and nanoparticles, as well as the morphologies of the various nanoparticles.

Properties	$c_{P}\;\left(J/kg\cdot K\right)$	k (W/m·K)	$\rho \;\left(kg/m^{3}\right)$	μ (kg/m·s)
H_2_O (water)	$4.179\times 10^{3}$	$613\times 10^{-3}$	$0.997\times 10^{3}$	$1\times 10^{-3}$
AlOOH(Boehmite alumina)	$0.618\times 10^{3}$	30	$6.05\times 10^{3}$	-

**Table 4:** The mesh-independence of the solution for different mesh numbers with a specific mesh number and size was studied by comparing the convective heat transfer coefficient and the pressure differential between the inlet and outlet of the HS.

Mesh	Number of Meshes	$h\;\left(W/m^{2}K\right)$	∆P (kPa)
M 1	1,014,534	8854	586
M 2	1,367,912	7842	545
M 3	1,791,400	7718	524
M 4	2,159,241	7689	515
M 5	2,455,901	7651	509
M 6	2,835,401	7591	508

**Table 5:** NF thermal conductivity with different NPs φ=0.06.

Shape NP	*k* (W/m K)	%Inc
Platelets 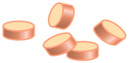	0.7089958	15.66
Blades 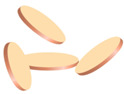	0.7137772	16.44
Cylinders 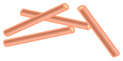	0.758281	23.70
Bricks 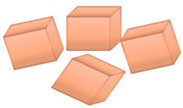	0.7369486	20.22
OS 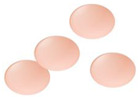	0.823751621	34.99

**Table 6:** NFs viscosity with different NPs φ=0.06.

Shape NP	μ (Pa s)	%Inc
Platelets 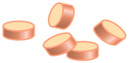	0.005448	443.14
Blades 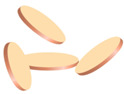	0.002327	132.00
Cylinders 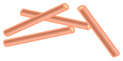	0.005081	406.58
Bricks 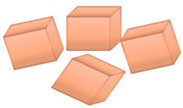	0.002819	181.05
OS 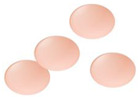	0.00125	24.62

## Data Availability

Not applicable.

## Abbreviation

**Nomenclature**	
C_p_	Specific heat (J/kg·K)
FOM	Figure of Merit
h	Heat transfer coefficient $\left(W/m^{2}\cdot K\right)$
HS	Heat sink
k	Thermal conductivity (W/m·K)
MHS	Micro heat sink
NF	Nanofluid
NP	Nanoparticle
p	Pressure (Pa)
PP	pumping power (W)
$q^{\prime \prime }$	Heat flux (W/m^2^)
$\dot{Q}$	volumetric flow (m^3^/s)
R	Thermal resistance (m^2^.K/W)
T	Temperature (K)
v	Velocity (m/s)
**Greek symbols**	
φ	Solid volume fraction
θ	Temperature uniformity (m^2^.K/W)
μ	Dynamic viscosity (kg/m·s)
ρ	Density ($\mathrm{kg}/m^{3})\;$
∆P	pressure difference
**Subscripts**	
Ave	Average
eff	Effective
f	Pure fluid
in	Inlet
m	Average fluid temperature
Max	maximum temperatures on the MHS
Mid	Average temperature of the MHS
Min	Minimum temperatures on the MHS
nf	Nanofluid
out	Outlet
P	Solid nanoparticle
